# GM-CSF Primes Proinflammatory Monocyte Responses in Ankylosing Spondylitis

**DOI:** 10.3389/fimmu.2020.01520

**Published:** 2020-07-16

**Authors:** Hui Shi, Liye Chen, Anna Ridley, Nancy Zaarour, India Brough, Cherilyn Caucci, Julia E. Smith, Paul Bowness

**Affiliations:** ^1^Nuffield Department of Orthopaedics, Rheumatology and Musculoskeletal Sciences, University of Oxford, Oxford, United Kingdom; ^2^Bioanalysis, Immunogenicity & Biomarkers, GlaxoSmithKline, Collegeville, PA, United States; ^3^Adaptive Immunity, GlaxoSmithKline, Stevenage, United Kingdom

**Keywords:** ankylosing spondylitis, monocyte GM-CSF, TNF-alpha, IL-23, CCL17

## Abstract

**Objectives:** GM-CSF is a pro-inflammatory cytokine with multiple actions predominantly on myeloid cells. Enhanced GM-CSF expression by lymphocytes from patients with Ankylosing Spondylitis (AS) has recently been described, however, its potential pathogenic role(s) in AS are unknown.

**Methods:** The effects of GM-CSF on TNF, IL-23, and CCL17 production by blood, PBMCs and isolated CD14+ monocytes from AS patients and healthy controls (HCs) were studied using ELISA. Serum CCL17 and GM-CSF and T cell GM-CSF production were studied in AS patients including pre-and on TNFi therapy.

**Results:** GM-CSF markedly increased TNF production by LPS-stimulated whole blood, peripheral blood mononuclear cells (PBMC) and purified monocytes from AS patients, with 2 h GM-CSF exposure sufficient for monocyte “priming.” Blocking of GM-CSF significantly reduced the production of TNF by whole blood from AS patients but not HCs. GM-CSF priming increased IL-23 production from LPS-stimulated AS and HC whole blood 5-fold, with baseline and stimulated IL-23 levels being significantly higher in AS whole blood. GM-CSF also stimulated CCL17 production from AS and HC blood and CCL17 levels were elevated in AS plasma. GM-CSF could be detected in plasma from 14/46 (30%) AS patients compared to 3/18 (17%) HC.

**Conclusion:** We provide evidence that GM-CSF primes TNF and IL-23 responses in myeloid cells from AS patients and HC. We also show CCL17 levels, downstream of GM-CSF, were elevated in plasma samples of AS patients. Taken together these observations are supportive of GM-CSF neutralization as a potential novel therapeutic approach for the treatment of AS.

## Introduction

Ankylosing spondylitis (AS) is a common inflammatory rheumatic disorder and a prototype of Spondyloarthritis (SpA), characterized by axial skeleton and sacroiliac joint involvement (1). AS affects ~0.3% of the UK population (2). Current therapies include NSAIDs, TNF inhibitors (TNFi) (3), and the IL-17A inhibitor secukinumab (4). However, an unmet therapeutic need remains for the significant proportion of patients who either do not tolerate or do not respond fully to these therapies.

GM-CSF is a key pro-inflammatory cytokine that possesses pleiotropic effects on myeloid cells including monocytes macrophages and dendritic cells augmenting innate and adaptive immune cell activation, amplifying tissue inflammation and contributing to the development of inflammatory diseases (5). There is growing evidence that GM-CSF is produced and active locally within inflamed tissues. Increased concentrations of GM-CSF have been reported in rheumatoid arthritis synovial fluid (RA) (6). We have shown that the blood and joints of AS and other axial SpA patients are enriched for GM-CSF-producing lymphocytes, and that the transcriptional signature of GM-CSF-producing CD4 T cells (“T_GM−CSF_”) overlaps but is distinct from that of classical IL-17A-producing TH17 cells (7).

Other pro-inflammatory cytokines and chemokines including TNF, IL-17, IL-23 CCL17, CCL22, and CXCL10 have been implicated in AS, (7, 8) with IL-23 capable of driving an animal model of spondyloarthritis (9). Monocytes are key producers of TNF, IL-23, and CCL17 and AS monocytes exhibit a more pro-inflammatory phenotype in than healthy control (10, 11).

We here study the role of GM-CSF in the pathogenesis of AS using patient-derived blood samples. We show that GM-CSF potentiates LPS-driven TNF and IL-23 production by cells from AS patients. We show that GM-CSF directly stimulates CCL17 production by AS patient blood and show elevated plasma CCL17 levels in AS patients that are reduced by TNFi therapy. Collectively, these data reveal potential roles of GM-CSF in the pathogenesis of AS and are supportive of the view that GM-CSF inhibition may be of therapeutic benefit in the treatment of AS.

## Materials and Methods

### Patient and Control Recruitment

Patients attending the Oxford University Hospitals National Health Service Foundation Trust were recruited. Blood samples were taken with informed written consent and their research use was ethically approved (Ethics reference number 06/Q1606/139, National Health Service, Health Research Authority, South Central—Oxford C Research Ethics Committee). Ankylosing Spondylitis (AS) patients met Assessment of Spondyloarthritis international society (ASAS) criteria for Axial Spondyloarthritis. Patients commencing TNFi therapy were deemed responders sustaining combined reduction of >4 points BASDAI and spinal pain. Patients with Rheumatoid Arthritis fulfilled 2010 ACR/EULAR criteria. Demographics of patients for plasma CCL17 comparison between AS, RA and HC are shown in Supplementary Table 1. Demographics of AS patients recruited for comparison of plasma CCL17 and T cell phenotyping between pre- and on TNFi treatment are shown in Supplementary Table 2. We have not specifically recorded the demographics of patients used for *in-vitro* GM-CSF –/+LPS stimulation experiments as inter-patient comparisons were not performed for these experiments (but all fulfilled ASAS diagnostic criteria for Axial SpA).

### Whole Blood Culture

One milliliter of whole blood from AS patients or healthy controls was incubated with 10 ng/ml human recombinant GM-CSF (PeproTech) or 10 μg/ml GM-CSF antibody (R&D) or IgG (R&D) at 37°C for 2 h, before being transferred into 2 ml tubes containing 1 ml of RPMI with or without 20 ng/ml LPS (LPS final concentration is 10 ng/ml, LPS is from Invivogen), and incubated at 37°C overnight. Supernatant was harvested for TNF, IL-23, or CCL17 ELISA.

### Cell Isolation

PBMCs were isolated from sodium-heparinized peripheral blood of AS patients or healthy control by density gradient centrifugation using Histopaque (sigma). Prior to isolation, whole blood was diluted 1:1 in RPMI 1640 medium (Gibco, Life Technologies, New York, NY, USA). CD14+ monocytes were isolated from PBMCs of AS patients or healthy control using CD14 microbeads (Miltenyi Biotec).

### Cell Culture

Pbmcs, CD14– PBMCs, or CD14+ monocytes were pre-incubated with 10 ng/ml human recombinant GM-CSF for 2 h, in 96-well plates (0.2 M per well), and incubated with LPS (10 ng/ml), at 37°C overnight. For GM-CSF priming condition, after 2 h incubation with GM-CSF (10 ng/ml), CD14+ monocytes were washed with PBS, and then stimulated with LPS and incubated overnight at 37°C. Pam3Csk4 (15 ng/ml), or RX848 (resiquimod 1 μg/ml) or CpGODN (10 μM) were also studied. ELISA was performed on supernatants according manufacturer's instructions; TNF, IL-23 both Thermo Fisher Scientific or CCL17 (R&D UK).

### RT^2^ Profiler PCR Array

CD14+ monocytes from AS and HC with or without GM-CSF treatment were assayed using the RT^2^ Profiler PCR array, designed in 384-wells for the detection of 84 JAK/STAT related genes. After 2 h of incubation with GM-CSF, CD14+ monocytes were pelleted and resuspend in TRIzol. Total RNA was isolated and extracted using an RNA extraction kit (Direct-zol RNA mini Prep, zymo research). After RNA extraction and quality assessment (NanoDrop) cDNA synthesis was performed using RT^2^ First Strand kit (Qiagen). Samples were prepared with the Master Mix and Template Cocktail and loaded into the 384–well RT^2^ Profiler PCR array. RT^2^ Profiler PCR arrays were run on ABI ViiA7 thermal cycler using the SYBR Green dye. ΔΔCt values determined. CT values greater than 35 were excluded. Fold change in expression of each gene in GM-CSF-treated cells was compared to untreated control. Genes with fold change ≥ 1.5 were considered up-regulated, and ≥ −1.5 as down-regulated.

### Intracellular Staining Flow Cytometry

Paired samples from 9 AS patients with active disease (BASDAI and spinal pain both >4) pre- and during TNFi treatment (3–6 months) were studied. PBMCs were isolated from fresh blood and cryopreserved at high cell density, 50–100 million/ml, in fetal bovine serum with 10% DMSO. This freezing condition was selected after comparing post-thawing viabilities of PBMCs frozen at different density and resulted in 80–90% viability. On the day of staining, PBMCs (isolated within 2 h of venesection and frozen in liquid nitrogen) were thawed and stimulated for 4 h at 37°C with phorbol myristate acetate (125 ng/ml; Sigma-Aldrich) and ionomycin (1 μg/ml; Sigma-Aldrich), in the presence of Golgiplug (Brefeldin A) and Golgistop (Monensin) (both from BD Biosciences). Staining antibodies and dilutions are shown in Supplementary Table 3. Surface staining was carried out on ice in the dark for 20 min. Cells were then fixed and permeabilized using Cytofix/Cytoperm solution (BD Biosciences) for 30 min and stained for intracellular cytokines. Flow cytometry was performed on a BD Fortessa calibrated daily with calibration and tracking beads from BD Biosciences. Data was analyzed using FlowJo software.

### CyTOF Staining

One microliter to one milliliter of 103Rh intercalator (Fluidigm) was added to cells and incubated for a 20 min at 37°C. Cells were resuspended in 50 μl staining buffer containing surface antibody master mix (antibodies used are listed in Supplementary Table 4) and incubated for 30 min at room temperature, then washed with MaxPar Cell Staining buffer. Cells were suspended in 500 μl of the 191Ir/193Ir intercalator and incubated overnight at 4^0^. Equalization beads (Fluidigm-Maxpar) were added at a concentration of 1:10 and the sample was acquired on a CyTOF-2 machine. Data analysis was carried out using Cytobank® software.

### Plasma Collection and GM-CSF Analysis

Plasma was obtained by centrifugation of fresh sodium heparin blood samples, and aliquots stored at −80 °C.

Levels of free GM-CSF were assessed using a GSK in house clinically validated Singulex bead-based immunoassay. Neat plasma samples were incubated in duplicate with beads bound with GSK3196165, an anti-GM-CSF monoclonal antibody, for 2 h. Beads were washed, and a fluorescent-labeled detection antibody was added and incubated for 1 h. Beads were washed again and the GM-CSF/fluorescent labeled complexes were eluted and transferred to a 384-well plate with buffer. The plate was read by the Singulex Erenna reader. GM-CSF concentrations were obtained by back-calculating signal data to a standard curve prepared with recombinant human GM-CSF in a filtered, low responding serum pool. Values below LLQ (lower level of quantification) were imputed to 0.5^*^LLQ. As this assay was validated for serum, parallelism between serum and plasma was demonstrated prior to testing plasma samples for this analysis (data not shown).

### Statistical Analysis

Prism was used for statistical analysis. Paired *t*-tests, unpaired *t*-tests, or one-way ANOVAs were used as denoted in the figure and Supplementary Figure legends. Statistical tests for RT^2^ Profiler PCR array experiment (**Figure 3E**) were performed using the QIAGEN data analysis website, *p* ≤ 0.05 was considered significant. The false discovery rate (FDR) was calculated using the Original FDR method of Benjamini and Hochberg. The CCL17 data presented in **Figure 4B** was not normally distributed and was therefore log-transformed prior to one-way ANOVA analysis.

## Results

### GM-CSF Potentiates TNF and IL-23 Production by LPS-Stimulated Whole Blood From AS Patients and HC

**Figure 1:**
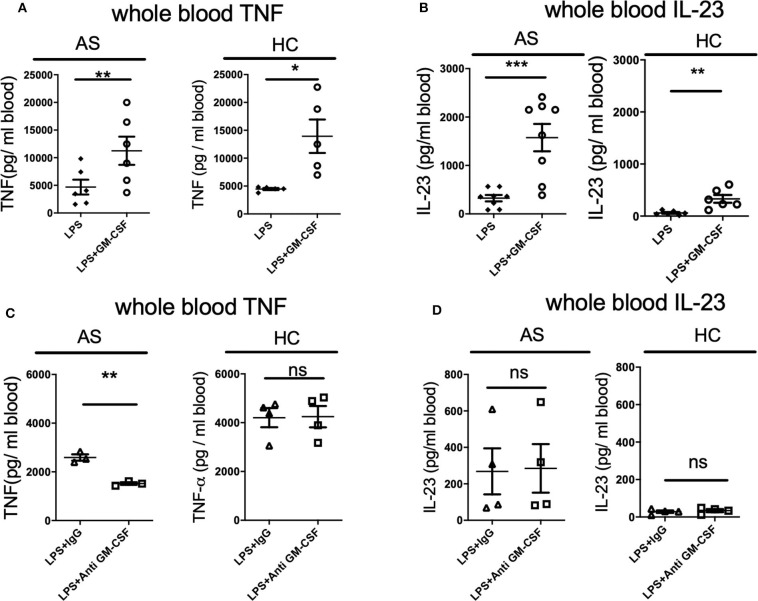
GM-CSF potentiates TNF and IL-23 production by LPS-stimulated whole blood from AS patients and healthy controls. Fresh whole blood from AS patients or healthy controls was treated with 10 ng/ml recombinant GM-CSF **(A,B)**, anti-GM-CSF or IgG control for 2 h **(C,D)**, then stimulated with 10 ng/ml LPS overnight. TNF **(A,C)** or IL-23 **(B,D)** measured in supernatants using ELISA. Data are represented as mean and SEM of independent donors in independent experiments. *p*-value was calculated using paired *t-*test. *p < 0.05, **p < 0.01, ***p < 0.001, ns: not significant. **(A)** AS *n* = 6, HC *n* = 5. TNF and IL-23 concentrations in the absence of LPS were less than 90 and 40 pg/ml respectively, AS *n* = 8, HC *n* = 6, **(C)** AS *n* = 3, HC *n* = 4, **(D)** AS *n* = 4, HC *n* = 4.

We first asked if GM-CSF regulates the whole blood response to the classical TLR4 agonist LPS in AS patient whole blood. Addition of GM-CSF significantly increased the production of TNF and IL-23 by LPS-stimulated whole blood from both AS patients and HC (Figures 1A,B). LPS stimulation of AS patient whole blood produced ~5-fold more IL-23 in the presence of GM-CSF (325.8 ± 187.1 vs. 1575.8 ± 798.1, *p* = 0.001, Figure 1B). For HC, lower levels of IL-23 were seen with LPS in the absence and presence of GM-CSF but with a similar 5-fold increase (61.3 ± 38.7 vs. 330.4 ± 183.3 *p* = 0.008). GM-CSF blockade reduced the production of TNF (but not IL-23) in response to LPS by whole blood from AS patients but not HCs (Figures 1C,D). These data show that GM-CSF potentiates the production of TNF and IL-23 in response to LPS by whole blood from both AS patients and HCs.

### GM-CSF Promotes the Production of TNF by Peripheral Blood Mononuclear Cells (PBMCs) From AS and HC, and Increases as PBMC IL-23 Production

To identify the cell type(s) responsible for the GM-CSF enhancement of cytokine responses to LPS, we separated peripheral blood into peripheral blood mononuclear cells (PBMCs) and non-PBMCs (containing granulocytes). Little TNF was produced in response to LPS by PBMC-depleted whole blood (data not shown). GM-CSF potently promoted the production of TNF by PBMCs from AS patients and HCs with LPS stimulation (Supplementary Figures 1A,B). LPS-induced IL-23 secretion was enhanced by the addition of GM-CSF to PBMCs from AS patients and a trend was observed for HCs (Supplementary Figure 1C,D). Thus, the effects of GM-CSF on TNF and IL-23 production are manifested by PBMCs.

### GM-CSF-Induced Potentiation of TNF Production by LPS-Stimulated PBMCs Is Contributed Primarily by CD14+ Monocytes

**Figure 2:**
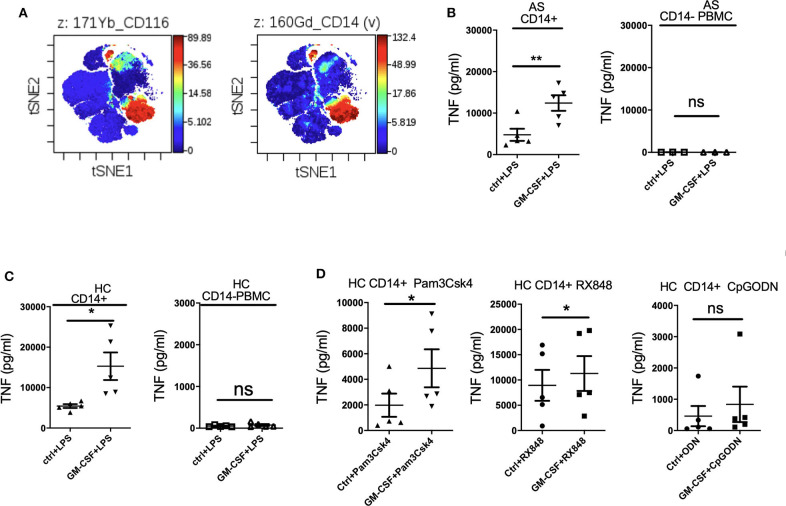
GM-CSF-potentiation of TNF production by LPS-stimulated PBMCs is contributed primarily by CD14+ monocytes. GM-CSF potentiates the response of monocytes to agonists for multiple TLRs. **(A)** GM-CSF receptorα(CD116) expression on CD14+ monocytes. CyTOF analyzed by viSNE clustering analysis of all live CD116 (GM-CSF Receptor α) events from PBMCs from a HC. Representative of *N* = 1. **(B,C)** CD14+monocyte (*n* = 5 AS patients and HCs) or CD14-depleted PBMCs (*n* = 3 AS patients) were treated with GM-CSF for 2 h followed by LPS (10 ng/ml) stimulation overnight. **(D)** CD14+ monocytes isolated from 5 HCs were treated with GM-CSF then with 15 ng/ml Pam3Csk4 or 1 μg/mlRX848 (resiquimod) or 10 μM CpGODN overnight. TNF levels in supernatant were determined by ELISA. Data are represented as mean and SEM of independent experiments, *p*-value was calculated using paired *t*-test, *p < 0.05, **p < 0.01, ns: not significant.

Mass cytometry (CyTOF) analysis confirmed high expression of GM-CSF receptor α (CD116) primarily on CD14+ monocytes (Figure 2A). We therefore hypothesized that GM-CSF likely regulates cytokine production by LPS-stimulated PBMCs primarily through monocytes. Indeed GM-CSF promoted TNF production by LPS-stimulated monocytes but not monocyte-depleted PBMCs from both AS patients and HC (Figures 2B,C). Thus, CD14+ monocytes are required for GM-CSF potentiation of LPS-stimulated TNF production by PBMCs. GM-CSF did not potentiate IL-23 production by isolated monocytes, implying that other cell type(s) are required for this effect (Supplementary Figure 2). However, within the LPS-stimulated PBMCs, monocytes were the principle producers of IL-23' with other leucocytes likely augmenting IL-23 production in the presence of GM-CSF (Supplementary Figure 3).

### GM-CSF Increases TNF Production by CD14+ Monocytes Stimulated With Multiple TLR Agonists

To investigate whether GM-CSF can affect TNF production by other TLR agonists, CD14+ monocytes were cultured with Pam3Csk4 (TLR1 and 2 agonist), RX848 (resiquimod, TLR7and 8 agonist), and CpGODN (TLR9 agonist). Figure 2D shows that GM-CSF enhanced both Pam3Csk4- and resiquimod-stimulated TNF production, indicating that GM-CSF can promote monocyte cytokine responses to multiple TLR agonists.

### Short Term (2 H) GM-CSF Exposure Is Sufficient to Enhance Cytokine Production by LPS-Stimulated Monocytes and Is Associated With Altered Expression of Multiple Cytokine Regulator Genes

**Figure 3:**
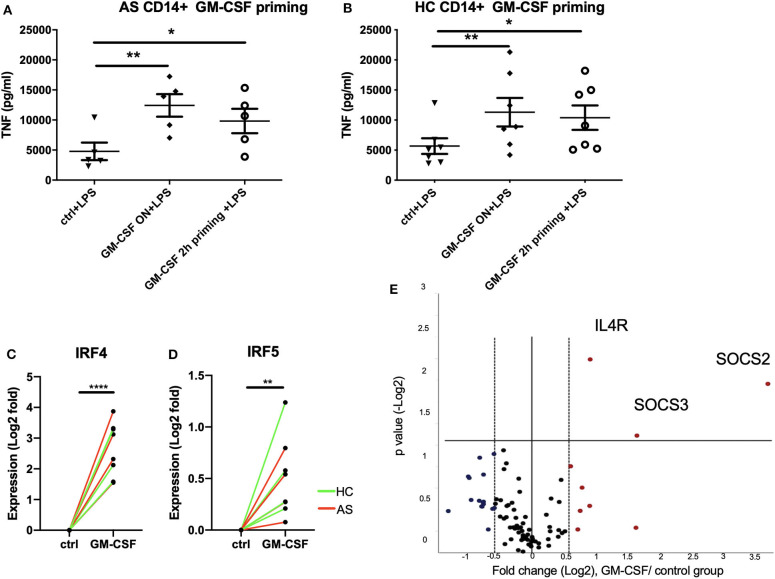
Short term (2 h) GM-CSF exposure enhances cytokine production by stimulated monocytes and is associated with altered expression of multiple cytokine regulators. **(A)** CD14+ monocytes were isolated from 5 AS patients or **(B)** 7 HCs. 1 million cells/ml CD14 monocytes were treated with GM-CSF for 2 h (and then washed) or overnight. Cells were then treated with LPS stimulation overnight. TNF level in supernatant was determined by ELISA. **(C,D)** Expression levels of IRF4 and IRF5 in GM-CSF-treated and untreated monocytes were measured using taqman qPCR. Red lines indicate AS patients, green lines indicate HCs. **(E)** CD14+ monocytes isolated from 4 AS and 4 HCs were treated with 10 ng/ml GM-CSF for 2 h. The effect of GM-CSF on genes in JAK/STAT signaling pathway was measured using the ProfilerRT2 PCR assay. The statistics for Profiler RT2 PCR array was carried out using QIAGEN data analysis website, *P* < 0.05 and fold change >1.5 was considered significant. Red dots indicate up-regulated genes, and blue dots indicate down-regulated genes. For **(A,B)**, data are represented as mean and SEM of independent donors, *P*-value was calculated using one way ANOVA and Dunnett's multiple comparison test. For **(C,D)**, *p*-value was calculated using paired *t*-test. *p < 0.05, **p < 0.01, ****p < 0.0001.

We next asked if short term exposure of GM-CSF is sufficient to increase the response of monocytes to stimulation. Two hours priming of monocytes with GM-CSF significantly promoted TNF production by LPS-stimulated monocytes from AS patients to levels similar to that seen with longer term GM-CSF exposure (Figure 3A). Similar effects were seen for monocytes from HC (Figure 3B). To investigate the mechanism we looked at expression of IFN regulatory factors 4 and 5 (IRF4 and IRF5) together with a panel of JAK/STAT genes RT-qPCR. IRF4 has been shown to mediate GM-CSF-driven cytokine production (12). Two hours GM-CSF priming is sufficient to up-regulate IRF4, IRF5, *IL4R, SOCS2*, and *SOCS3* in CD14+ monocytes (Figures 3C–E, FDR for *IL4R, SOCS2*, and *SOCS3* were 0.0073, 0.0297, and 0.0017, respectively).

### GM-CSF Stimulates CCL17 Production From AS and Control Whole Blood. CCL17 Levels Are Elevated in AS Patients' Plasma and Are Reduced With TNFi Therapy

**Figure 4:**
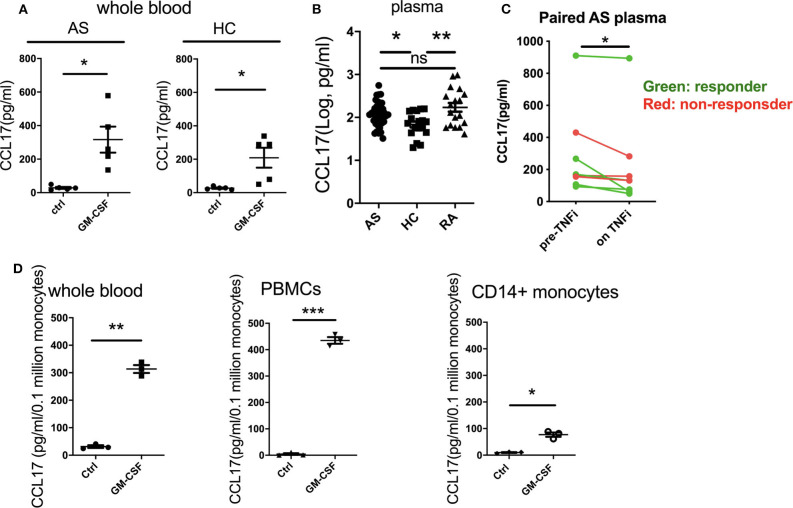
GM-CSF increases the production of CCL17 by whole blood from AS and HC. CCL17 is elevated in AS plasma and is reduced after TNFi treatment. Whole blood from AS patients or healthy controls was treated with 10 ng/ml recombinant GM-CSF for 2 h **(A)**, then cultured overnight. Supernatant was measured for CCL17 using ELISA. Data are represented as mean and SEM of independent donors, *p*-value was calculated using paired *t*-test. *p < 0.05. **(A)** AS, HC *n* = 5. **(B)** Plasma CCL17 levels from 36 AS, 18 HC and 18 RA. For AS, 18 of them were biologics naive and 18 of them were on biologics. *P*-value was calculated using one-way ANOVA on the log-transformed data. **(C)** 8 paired pre- and on TNFi treatment AS plasma samples were collected, CCL17 in plasma was determined by ELISA. Red dots show TNFi non-responders, and green dots show TNFi responders. **(D)** Whole blood, PBMCs, or CD14+ monocytes (*n* = 3) from healthy controls was treated with 10 ng/ml recombinant GM-CSF then cultured overnight. Supernatant was collected and measured for CCL17 production using ELISA. Data was normalized as pg/ml/0.1 million CD14+ monocytes. Data are represented as mean and SEM of independent donors, *p*-value was calculated using paired *t*-test. *p < 0.05, **p < 0.01, ***p < 0.001, ns: not significant.

Recent studies have shown that GM-CSF can promote CCL17 production via IRF4 gene induction (12). We therefore investigated the effect of GM-CSF on CCL17 production in AS and HC whole blood. GM-CSF increased CCL17 levels in whole blood from AS and HC (Figure 4A). This increase was ~11-fold for the AS patients, from 29 to 317 pg/ml, and 8-fold for the HC, from 27 to 209 pg/ml. We next investigated if CCL17 levels were elevated in plasma from AS patients compared to HC and RA patients. Patients with either AS or RA were shown to have higher plasma levels of CCL17 compared to HC (Figure 4B). The backtransformed mean plasma CCL17 levels were 120 pg/mL (95% CI: 93.0 to 154), 68.5 pg/mL (95% CI: 47.9 to 98.0), and 172 pg/mL (95% CI: 120 to 246) for AS, HC, and RA patients respectively. We next studied a second AS patient cohort about to commence therapeutic blockade of TNF (TNFi) and, although numbers were small, observed reduced CCL17 levels upon TNFi therapy (Figure 4C).

We then asked if GM-CSF regulated PBMC CCL17 production directly through monocytes. Whilst GM-CSF indeed induced CCL17 release from isolated monocytes, comparison with experiments using whole blood and PBMCs suggested that other cell types may potentiate this effect (Figure 4D). To determine whether cell types in addition to monocytes produced CCL17 in response to GM-CSF, we performed intracellular staining of CCL17 using GM-CSF-stimulated PBMCs. Only CD14+ monocytes (but not CD14- cells) secreted CCL17 in response to GM-CSF treatment (Supplementary Figure 4). Of note, LPS stimulated completely abolished CCL17 production (Supplementary Figure 4). In summary GM-CSF-induced CCL17 production by PBMC is almost entirely from CD14+ monocytes but is enhanced in the presence of other cell types.

### AS Patients Responsive to TNFi Therapy Have Elevated Pretreatment Numbers of GM-CSF-Producing CD8+ T Cells

GM-CSF-producing lymphocytes in paired PBMC samples from AS patients pre- and on TNFi treatment were quantified using intracellular staining flow cytometry. Although numbers were small (9 pairs), TNFi responders had a higher baseline percentage of GM-CSF-producing CD8 T cells than TNFi non-responders (Supplementary Figure 5). TNFi treatment did not affect the frequency of GM-CSF-producing CD4 or CD8 T cells.

### GM-CSF Levels in Plasma From AS Patients Pre- and on TNFi

GM-CSF was detected by Singulex bead-based immunoassay in 3/18 HCs, 2/18 RA patients, 4/20 AS patients naïve for biologic treatment, 5/18 AS patients on biologic treatment, and 5/8 AS patients studied both pre- and on TNFi treatment, totalling 14/46 (30%) AS patients compared to 3/18 (17%) HC (Supplementary Table 5). GM-CSF levels ranged from 0.87 to 82.39 pg/ml. Overall no significant differences were observed between the groups studied (Supplementary Figure 6).

## Discussion

We have previously described that patients with axial SpA have increased numbers of GM-CSF-secreting lymphocytes (7). We here describe potential mechanisms for roles of GM-CSF in AS pathogenesis. We show that GM-CSF potentiates the production of TNF by LPS-stimulated whole blood, PBMCs and monocytes from AS patients and healthy controls. Monocytes were required for this effect, which was also seen for TLR1/2 or TLR7/8 agonists. Furthermore, neutralizing GM-CSF significantly reduced the production of TNF by AS patient blood but not healthy controls. Notably 2 h GM-CSF exposure was sufficient to potentiate LPS-stimulated monocyte TNF production, and this exposure was associated with up-regulation of IRF4, IRF5, and other genes involved in cytokine regulation.

**Figure 5:**
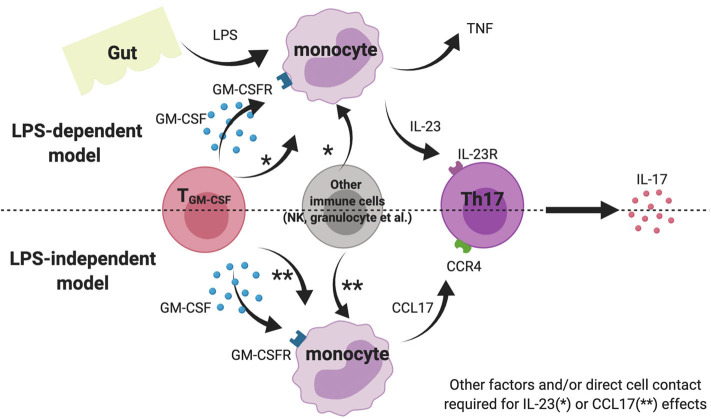
Cartoon model suggesting possible role of GM-CSF and monocytes in AS pathogenesis. Note this inflammation amplification may occur in gut-associated lymphoid tissue, blood or within the joint.

We also show that GM-CSF potentiates IL-23 production by LPS-stimulated whole blood and PBMC from AS patients. This effect was not seen for isolated AS monocytes, implying involvement of other cell types. Lastly, we demonstrate that GM-CSF stimulates CCL17 production from AS and HC blood; CCL17 levels were elevated in AS plasma and, in a small cohort of patients, were reduced upon TNFi therapy. A cartoon model showing the potential pro-inflammatory the roles of GM-CSF in SpA is shown in Figure 5.

AS is characterized by chronic (often subclinical) gut inflammation and Reactive Arthritis is a related form of SpA that often follows gastrointestinal infection. Both these scenarios might lead to increased gastrointestinal or systemic LPS exposure. Indeed AS patients have recently been reported to have impaired gut vascular barrier and increased circulating LPS levels (13). Whilst prior LPS exposure can “tolerize” monocytes to future stimulation, we propose that the presence of GM-CSF may prevent or have the opposite effect, driving monocyte “training” to enhance subsequent cytokine responses. Such an effect has recently been shown for S100 proteins in inflammatory arthritis (14).

The current study provides evidence that AS patients' monocytes are indeed “primed” to produce high levels of TNF upon LPS exposure. It was likely that GM-CSF contributed to this effect endogenously, as LPS-stimulated TNF levels were reduced by anti-GM-CSF antibody treatment *ex vivo* in whole blood from AS patients but not healthy controls. This AS-specific effect of anti-GM-CSF strongly supports the increased levels of GM-CSF in AS blood and the therapeutic potential of blocking GM-CSF in AS. GM-CSF was also shown to potentiate LPS-stimulated IL-23 levels in whole blood from AS patients and HC by ~5-fold. Interestingly the levels of IL-23 stimulated by LPS were always significantly greater in AS patients compared to HC, whether GM-CSF was exogenously applied or not. We decided to investigate IL23 levels in these experiments as dysregulation of the IL-23/IL-17 pathway has been associated with AS pathology (9, 15, 16).

It is interesting that the addition of GM-CSF enhanced the production of LPS-induced IL-23 by whole blood and PBMCs, but not by isolated CD14+ monocytes. It is likely therefore that other cell types such as T cells are required for this effect, which might require direct cell contact or additional soluble factors. We also show here that short term priming (2 h) of monocytes with GM-CSF is sufficient to enhance TNF cytokine production by LPS-stimulated monocytes. GM-CSF priming altered the transcriptome of monocytes and regulated the expression of IRF4, IRF5, and other cytokine regulatory genes, confirming and extending previous findings, with IRF4 shown previously to drive CCL17 production in human monocytes stimulated overnight with GM-CSF (12). The “GM-CSF potentiation effect” was also observed in our studies for TLR 1/2 or TLR7/8 agonists and are supportive of a role for monocytes and/or monocyte-derived cells in AS pathogenesis, where alterations in GM-CSF expressing cell compartments (e.g., T-cells, NK cells, ILCs) (7) might contribute to driving aberrant or excessive inflammatory responses. This view is supported by findings in our study that suggest (although the patient numbers were small) that AS patients responding to TNFi therapy appeared to have a higher percentage of GM-CSF- producing CD8 T cells than TNFi non-responders.

GM-CSF has been shown to drive CCL17 expression and protein release from human monocytes via JMJD3 (a histone demethylase) and IRF4 and that absence/inhibition of these mediators ameliorates inflammatory arthritis and pain in mouse models (12). We here show for the first time that levels of CCL17 in whole blood from AS patients and healthy controls could be elevated by incubation with GM-CSF *ex vivo*. We also show that baseline CCL17 levels in AS plasma were elevated compared to healthy controls, and CCL17 levels may be reduced following anti-TNF treatment. Although our prospective cohort was small, our findings agree with two previous studies (17, 18). Additionally, we found that LPS stimulation abolished the production of CCL17 by monocytes, suggesting CCL17 more likely exhibits its biological function in the chronic LPS-independent model rather than acute inflammatory scenario (Figure 5).

It is interesting that blocking of GM-CSF reduced the LPS-induced TNF-alpha production in AS but not healthy control whole blood, and did not affect LPS-induced IL-23 production in AS or HC. A possible explanation is that whilst AS patients have higher concentrations of GM-CSF than healthy controls, the level is lower than that required for the enhancement of IL-23 production. Notably, lymphocytes from inflamed synovium have been shown to produce more GM-CSF than those from peripheral blood, and might result in tissue-localized high concentrations. Thus, it is plausible to predict that GM-CSF inhibition might reduce IL-23 as well as TNF production in inflamed joints and consequently provide additional therapeutic benefits over and above TNF inhibition alone.

Circulating levels of CCL17 have been shown to be suppressed in RA subjects in the clinic following blockade of the GM-CSF receptor alpha subunit, and CCL17 was proposed to serve as a specific pharmacodynamic marker for GM-CSF pathway targeting (19). Our data suggest that in AS, CCL17 could also prove a potentially important mediator regulated by GM-CSF and support recent work by Cook et al. (20). We note that the GM-CSF–CCL17 signaling axis can be downstream of TNF and that CCL17 has recently been postulated as a therapeutic target in other forms of arthritis (21).

## Conclusions

Our data support an important role of GM-CSF in AS, suggesting pathogenic mechanisms that are likely active in AS including promotion of LPS-stimulated TNF and IL-23 cytokine release and direct effects on CCL17 levels. We also show CCL17 levels were elevated in plasma samples of AS patients. Taken together these observations are supportive of GM-CSF neutralization as a potential novel therapeutic approach for the treatment of AS. The efficacy of namilumab, a GM-CSF neutralizing monoclonal antibody, is to be assessed in a Phase II Proof-of-Concept clinical study in moderate-to-severe axial spondyloarthritis patients NCT03622658.

## Data Availability Statement

All datasets generated for this study are included in the article/Supplementary Material.

## Ethics Statement

The studies involving human participants were reviewed and approved by South Central - Oxford C Research Ethics Committee. The patients/participants provided their written informed consent to participate in this study.

## Author Contributions

HS, LC, AR, IB, CC, and NZ performed the studies and take complete responsibility for the integrity of the data and the accuracy of the data analysis. PB, HS, and JS designed the experiments and had full access to all the data. PB and HS wrote the original draft of the manuscript. All authors reviewed and edited the manuscript and approved the final version. All authors contributed to the article and approved the submitted version.

## Conflict of Interest

The authors declare that this study received funding from GlaxoSmithKline (GSK). The funder had the following involvement with the study: partnering on analysis of samples, experimental design, interpretation of data, pre-submission review of this article and the decision to submit it for publication. CC and JS declare they are employees and stockholders of GSK.
