# Supplementary material for: GM-CSF Primes Proinflammatory Monocyte Responses in Ankylosing Spondylitis
Source: Front Immunol. 2020 Jul 16;11:1520. doi: 10.3389/fimmu.2020.01520 (PMC7378736; doi:10.3389/fimmu.2020.01520)
Supplement: Supplementary file 1 [file Data_Sheet_1.PDF]

SUPPLEMENTARY DATA for Shi et al

**Supplementary Table 1. Demographics of AS, RA patients and healthy controls recruited for measurement of plasma CCL17**			
	Ankylosing Spondylitis (n=38)	Rheumatoid Arthritis (n=18)	Healthy controls (n=18)
Age, mean (range) years	48 (29-72)	62.4 (41-91)	44.9 (23-60)
Sex, male/female	25/13	4/14	9/9
HLA-B27+ no. (%)	26* (76.5%)	n/a	n/a
BASDAI, mean (range)	3.48 (0-10)	n/a	n/a
DAS28-CRP, mean (range)	n/a	3.3 (0.1-5.7)	n/a
DMARD therapy, current (previous)	4 (0)	18 (0)	n/a
TNFi therapy, current (previous)	18 (2)	4 (0)	n/a
CRP, mean (range)	5.7 (0.3-31.3)	5.2 (0.2-13.7)	n/a
*unknown HLA-B27 status in 4 AS patients			

**Supplementary Table 2. Demographics of paired pre- and on TNFi AS patient cohort (n=11, GM-CSF-producing T cells studied in 9, plasma GM-CSF in 8)**	
	Ankylosing Spondylitis
Age, mean (range) years	43.5 (24-65)
Sex, male/female	6/5
HLA-B27+ no. (%)	10* (100%)
TNFi Responders/non-responders	7/4
Pre BASDAI, mean (range)	5.4 (3.2-7.3)
Post BASDAI, mean (range)	3.3 (0.7-6.2)
Pre VAS spinal pain, mean (range)	6.3 (3.1-8.7)
Post VAS spinal pain, mean (range)	4.1 (0.5-7.5)

*unknown HLA-B27 status in 1 AS patient

**Supplementary Table 3. FACS staining panel for immunophenotyping PBMC**			dilution	µL Ab
Surface	Supplier	Cat. No.		
L/D eF780	eBioscience	65-0865-14	250	0.2
CD3 BV785	BioLegend	317330	50	1
CD4 APC	BioLegend	300537	50	1
CD8a BV510	BioLegend	301048	50	1
TCR Vδ2 PE	BioLegend	331408	25	2
CD56 BV711	BD	563169	50	1
CD161 BV421	BioLegend	339914	25	2
CD127 BV605	BioLegend	351333	50	1
ICS				
IFNγ AF700	BioLegend	506516	100	0.5
IL-17A FITC	eBioscience	11-7179-42	50	1
GMCSF PerCPCy5.5	BioLegend	502312	50	1
IL-22 PE/Cy7	eBioscience	25-7229-42	50	1

Supplementary Table 4. CYTOF staining panel for immunophenotyping PBMC

Marker	Metal	Clone	Concentration (ul/stain)
CD45	89Y	2B11	0.5
CD19	Nd142	HIB19	1
CD14	Gd160	RM052	1

HLA-DR	Yb174	YE2/36HLK	1
CD3	Er170	UCHT1	1
CD116	Yb171	4H1	1

Supplementary Table 5. GM-CSF concentrations (pg/ml) in plasma from healthy controls, Ankylosing Spondylitis and Rheumatoid Arthritis patients						
					AS Patients with paired samples	
**Subject**	**Healthy**	**RA**	**Naïve AS**	**AS on Biologics**	**Pre-TNFi Treatment**	**On TNFi Treatment**
**1**	BLQ*	BLQ	BLQ	BLQ	BLQ	BLQ
**2**	BLQ	BLQ	4.01	BLQ	BLQ	BLQ
**3**	5.27	BLQ	BLQ	BLQ	BLQ	25.5
**4**	BLQ	BLQ	1.09	BLQ	2.62	8.91
**5**	BLQ	BLQ	BLQ	BLQ	37.5	2.04
**6**	BLQ	BLQ	BLQ	BLQ	BLQ	BLQ
**7**	BLQ	BLQ	BLQ	BLQ	0.87	1.35
**8**	BLQ	BLQ	BLQ	BLQ	2.89	BLQ
**9**	BLQ	BLQ	BLQ	0.93		
**10**	BLQ	2.66	BLQ	BLQ		
**11**	BLQ	BLQ	BLQ	2.94		
**12**	1.67	BLQ	19.95	60.25		
**13**	BLQ	BLQ	BLQ	BLQ		
**14**	8.02	82.39	BLQ	BLQ		
**15**	BLQ	BLQ	BLQ	BLQ		
**16**	BLQ	BLQ	BLQ	BLQ		
**17**	BLQ	BLQ	BLQ	2.29		
**18**	BLQ	BLQ	1.42	10.53		
**19**			BLQ			
**20**			BLQ			
**# of Subjects Measured/Total**	3/18	2/18	4/20	5/18	4/8	4/8
**Total Subjects Measured**	22/90					

*BLQ: Below the assay's limit of detection: ie <0.781pg/ml

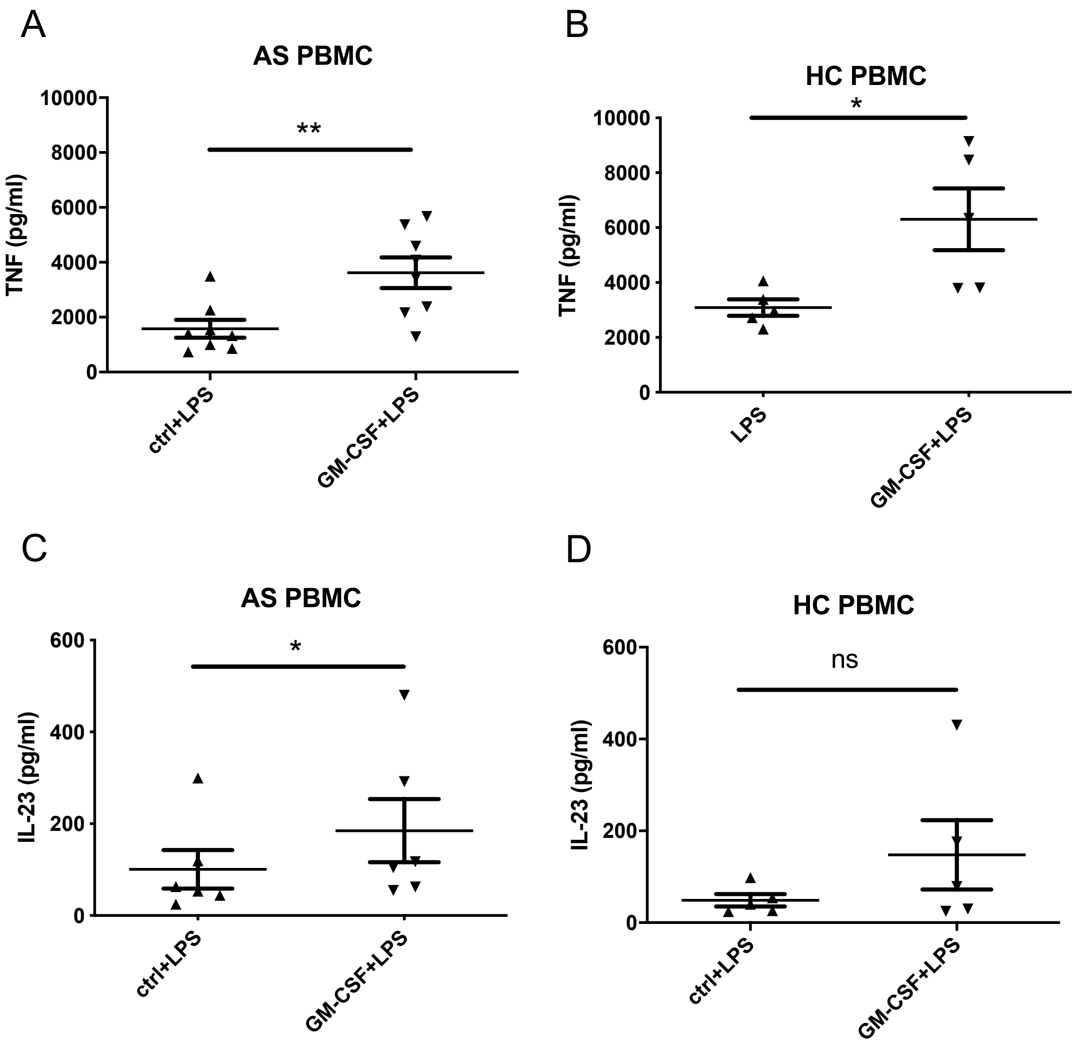

Supplementary Data Figure 1. GM-CSF promotes the production of TNF by AS and HC peripheral blood mononuclear cells (PBMC) and of IL23 by AS PBMC. PBMCs (1 million/ml) from AS patients (**A** and **C**) or healthy controls (**B** and **D**) were treated with 10ng/ml recombinant GM-CSF for 2 hours, then stimulated with 10 ng/ml LPS overnight. Supernatants were measured for TNF (**A** and **B**) or IL-23 (**C** and **D**) by ELISA. Data are represented as mean and SEM of independent donors/experiments, p value was calculated using paired t test. * $p < 0.05$, ** $p < 0.01$. A. n=8, B n=5, C. n=6, D. n=5.

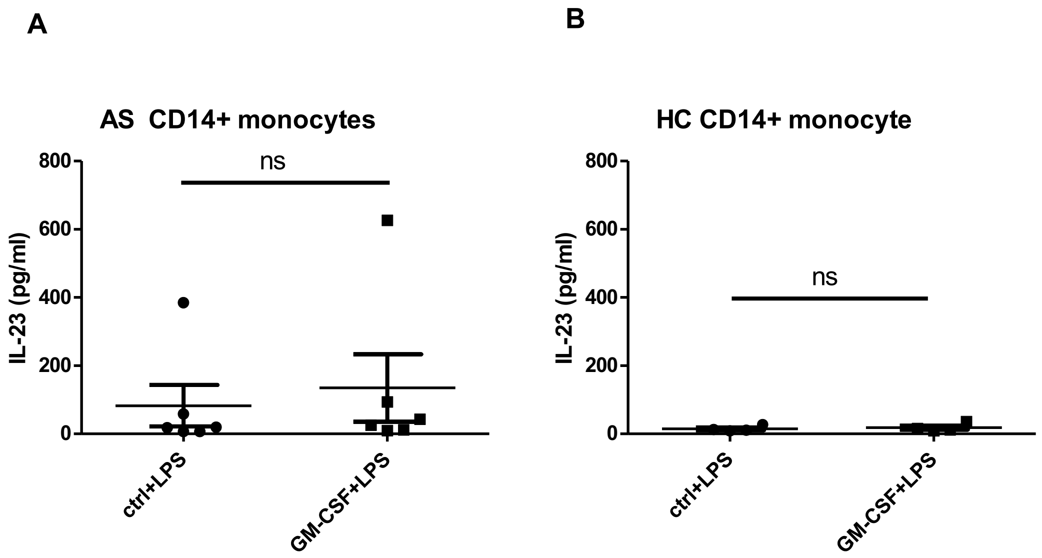

Supplementary Data Figure 2. GM-CSF does not promote the production of IL-23 by LPS-induced CD14+ monocytes from AS patients (n=6) or HCs (n=4). IL-23 level in supernatant was determined by ELISA. Data are represented as mean and SEM of independent donors/experiments, p value was calculated using paired t-test, ns: not significant.

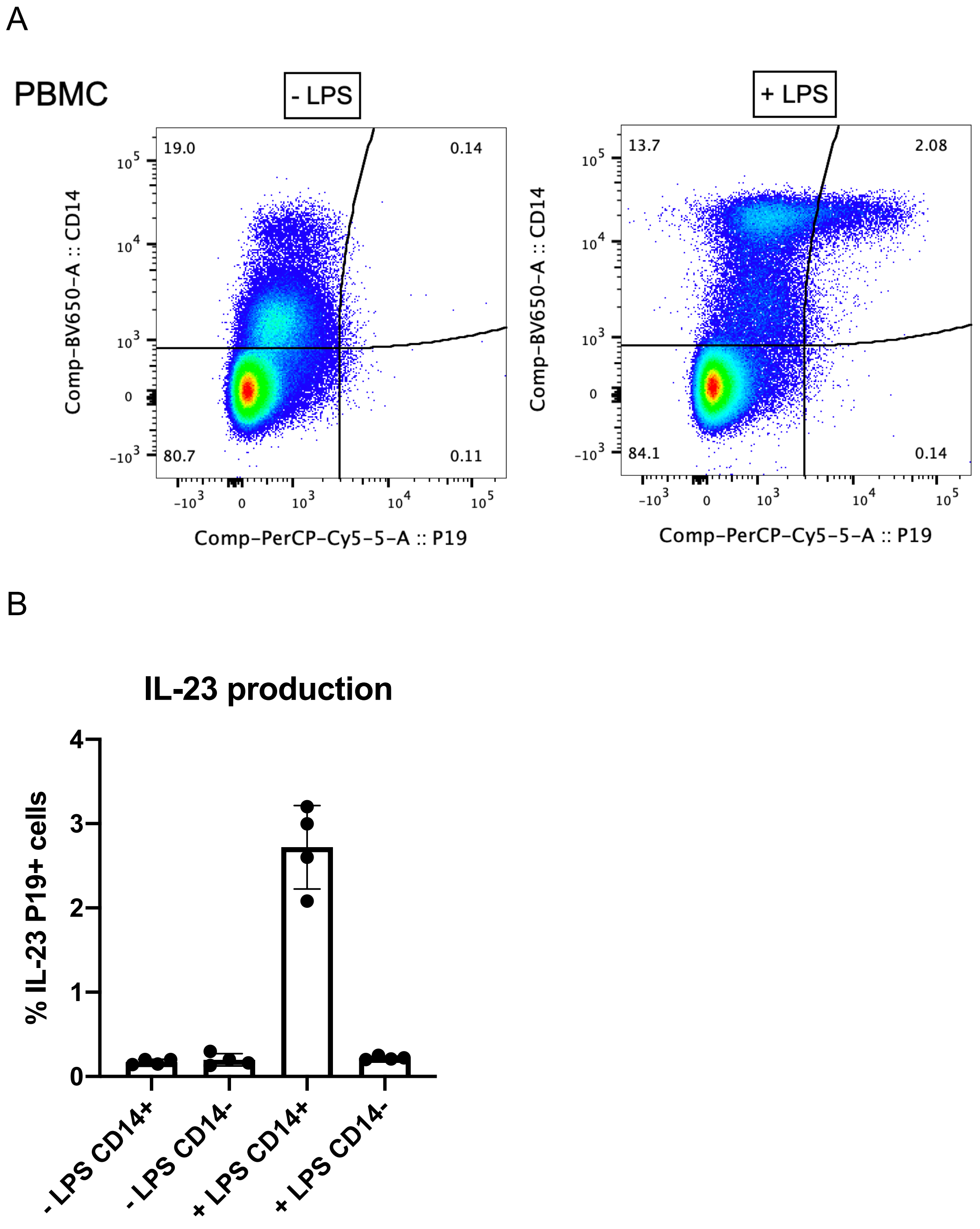

Supplementary Data Figure 3. CD14+ monocytes are the principle producers of IL-23 following LPS stimulation of PBMC. Freshly isolated PBMCs from 4 healthy control were cultured with LPS overnight. Brefeldin A was added 12 hours before harvest of cells for intracellular staining of the IL-23-specific P19, gating on CD14+ or CD14- leucocytes. **(A)** representative flow cytometry plot. **(B)** composite data of 4 HCs.

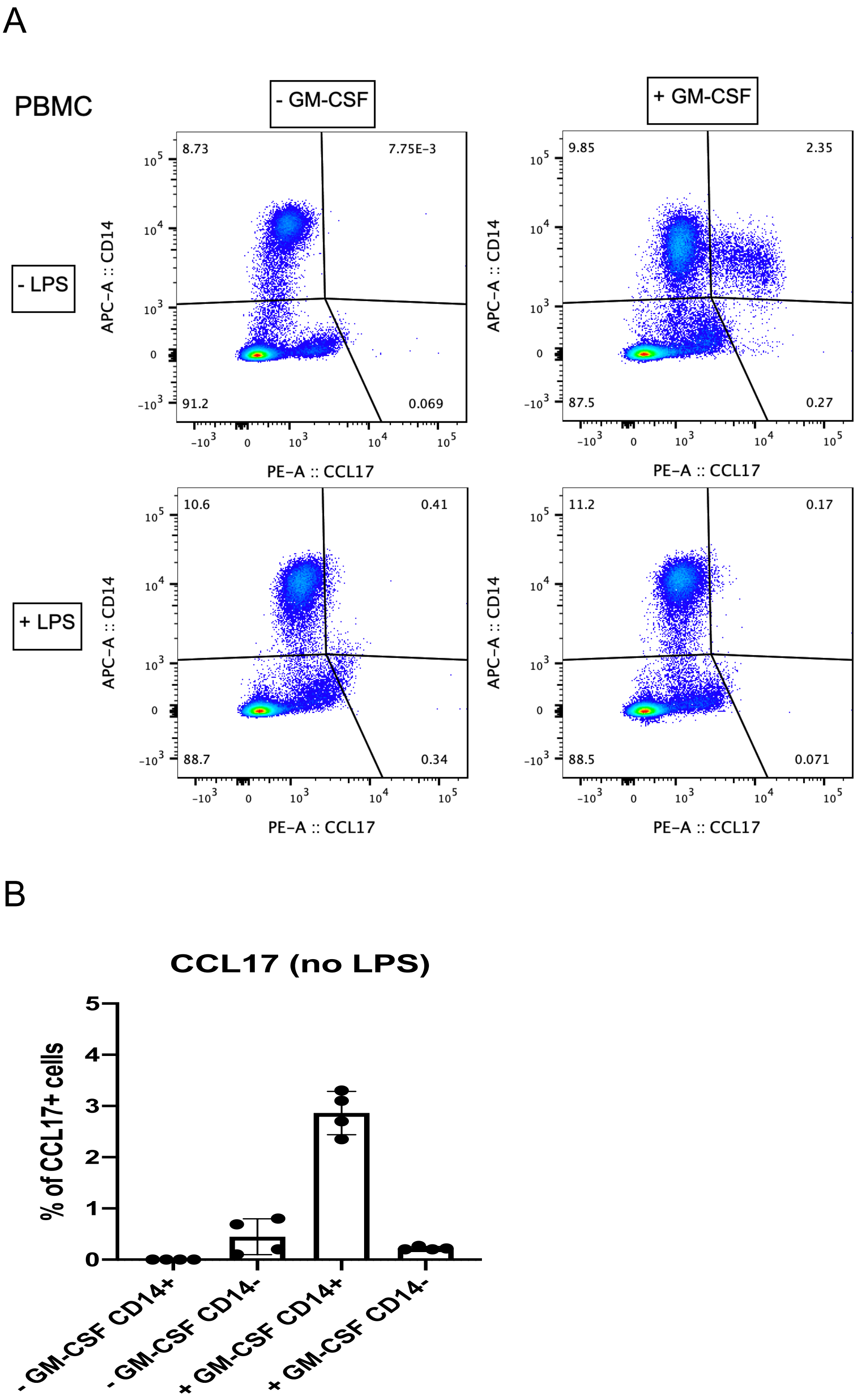

Supplementary Data Figure 4. GM-CSF-induced CCL17 production by PBMC is contributed by CD14+ monocytes and abolished by LPS stimulation. Freshly isolated PBMCs from 4 healthy controls were cultured with GM-CSF overnight with or without LPS stimulation. Brefeldin A was added to PBMCs 4 hours before harvest of cells for intracellular staining of

CCL17. **(A)** representative flow cytometry plot. **(B)** composite data of 4 HCs in the absence of LPS stimulation.

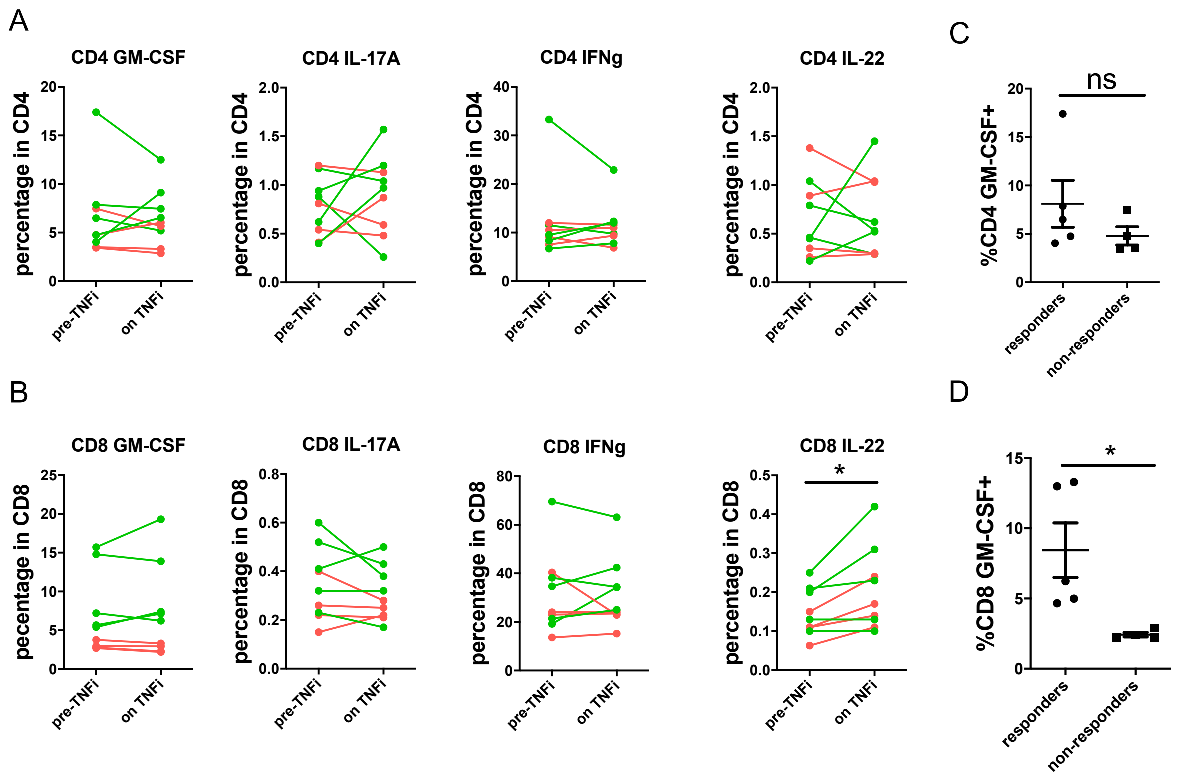

Supplementary Data Figure 5. Frequencies of GM-CSF and other cytokine-producing CD4 and CD8 T cells in the peripheral blood of AS patients pre- and on TNF blockade. ICS-FACS gated on live CD3+ lymphocytes from paired pre-and on TNFi PBMC samples from 9 AS patients. The percentage of cytokine (GM-CSF, IL-17A, IFN-g and IL-22) producing CD4 (**A**) and CD8 (**B**) are shown. Paired data for each independent donor are represented. p value was calculated using paired t test.* $p < 0.05$. Red dots show non-responders, and green dots show responders to TNFi therapy (combined BASDAI and spinal pain reduction of >4 points). The percentage of GM-CSF-producing CD4 (**C)** and CD8(**D)** from pre-TNFi samples was compared between responders (n=5) and non-responders (n=4), p value was calculated using t test.* $p < 0.05$. Analyses not corrected for multiple comparisons.

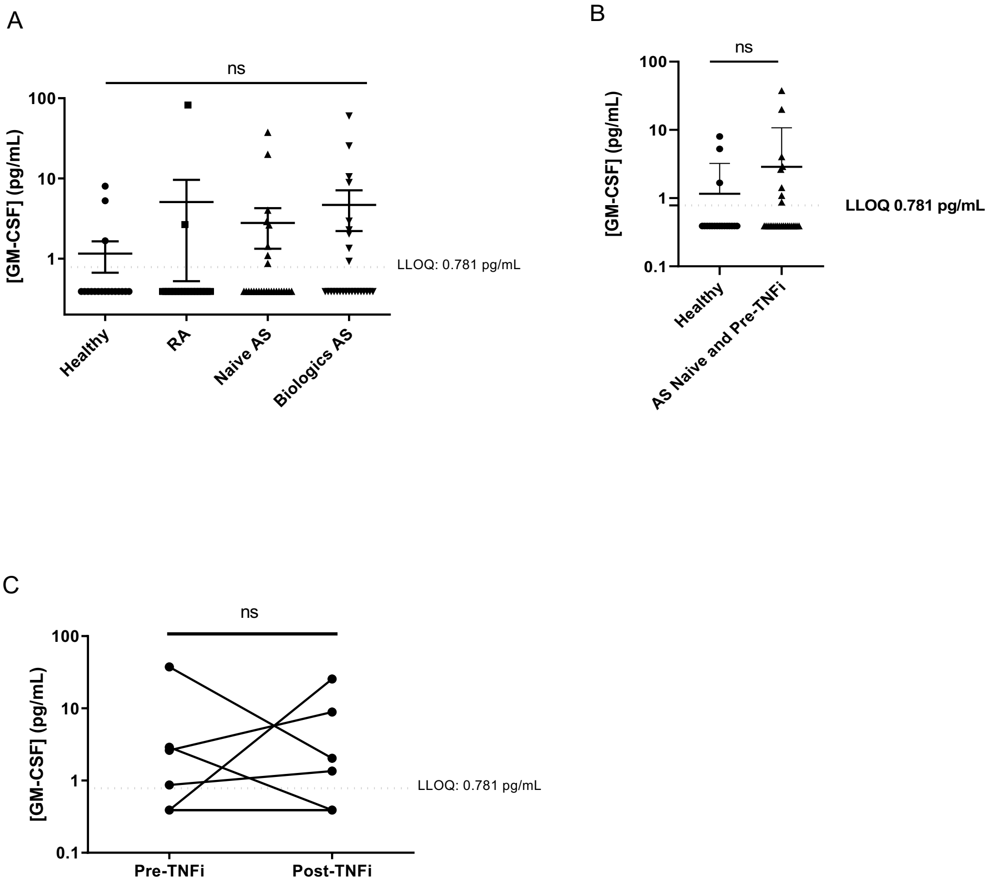

Supplementary Data Figure 6. GM-CSF can be detected in some plasma samples from AS HC and RA patients. (A) 54 AS, 18 HC and 18 RA plasma samples were collected. Among 54 AS, 28 of them were naïve or pre-TNFi and 26 of them were on biologics or post-TNFi. **(B)** 18 healthy shown in A are compared with pooled TNFi naïve and pre-TNFi AS patients shown in A. **(C)** 8 paired pre and on TNFi treatment AS plasma. GM-CSF levels in plasma were determined by bead-based immunoassay on the Singulex platform. P values were calculated using One-way ANOVA for A, t-test for B and paired t test for C. No significant difference observed between healthy and disease or pre- and on TNFi.
